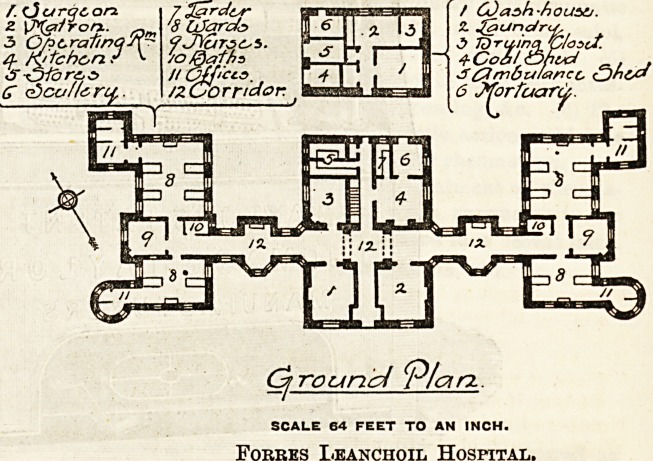# Forres Leanchoil Hospital

**Published:** 1892-09-10

**Authors:** 


### HOSPITAL CONSTRUCTION.

### FORRES LEANCHOIL HOSPITAL.

This Cottage Hospital owes its existence mainly to the munificence of Sir Donald A. Smith, K.C.M.G., of Montreal,

SCALE 64 FEET TO AN INCH.

Forres Leanchoil Hospital.

Canada, a native of Forres. It is intended for the poor of Forres and the surrounding district, the population of which is about 10,000; of this about 5,000 belongs to the town.

The plan shows a central block, with two wings connected with the central block of corridors, and a small detached building at the rear of the central block.

The central block is two storeys in height, and contain on the ground floor the main entrance, with Matron's and surgeons' rooms on either side; operation room, kitchen, scullery, larder, and stores behind; on the upper floor are bed-rooms for the Matron, nurses, and servants.

In connecting corridors between the central block and the wards are widened out in the centre, each with a bay window and a fireplace recess to form day-rooms for convalescent patients.

The wings each contain two wards, one for four beds, the other for two beds, with a nurse's room and bath-room placed between the two. The larger wards are 24 feet long by 20 feet wide, and 13 feet high, giving a superficial area of 120

feet, and cubic space of 1,560 feet per bed. The smaller wards are 13 feet long by 20 feet wide, and give a superficial space of 130 feet, and a cubic space of 1,690 feet per bed. The wards are lighted by windows in the side and end walls, fitted with double-hung sashes with fanlights over.

The wards are provided with ventilating stoves, especially designed for this building, supplied with fresh air from the outside, and the corridors are warmed by "Galton" stoves.

For ventilating the wards, in addition to the windows, air shafts are fixed in the walls, fitted with adjustable hopper arrangements, at a height of 10¾ feet from the floor. These hoppers, of which there is one to each bed, can be opened or closed at will. Exit ventilators for foul air are provided in the roof.

The ward floors are laid with hard Canadian maple, in 3-in. widths, wax polished, and laid on deal counter flooring on wooden joists.

The small detached building contains a wash-house and laundry, ambulance house, and mortuary.

The architect of the hospital was Mr. H. Saxon Snell, of London.

## Figures and Tables

**Figure f1:**